# Primary pulmonary artery sarcoma treated with simultaneous pulmonary valve replacement, artificial vessel replacement, and total right pneumonectomy: a case report

**DOI:** 10.1186/s44215-025-00221-6

**Published:** 2025-08-27

**Authors:** Kento Fujii, Hideki Morita, Hiroshi Nagano, Hiroyuki Kawaura, Hidetaka Kariya, Minoru Kowada, Takehiro Shirasugi

**Affiliations:** 1 Department of Cardiovascular Surgery, Saitama Red Cross Hospital, Saitama, Japan; 2 Department of Cardiovascular Surgery, Disaster Medical Center, Tokyo, Japan; 3 Department of Emergency, Saitama Red Cross Hospital, Saitama, Japan

**Keywords:** Primary pulmonary artery sarcoma, Pulmonary valve replacement, Total right pneumonectomy, Gene panel test

## Abstract

**Background:**

Primary pulmonary artery sarcoma progresses extremely rapidly and has a poor prognosis. This condition is managed with surgical resection and multimodality therapy. However, standardized treatment is not available.

**Case presentation:**

A 54-year-old woman was brought to the emergency department because of chest pain and worsening dyspnea that had developed in the past month. Contrast-enhanced computed tomography scan revealed severe stenosis extending from the pulmonary artery trunk to the right pulmonary artery due to an embolic substance. Because primary pulmonary artery sarcoma was suspected, emergency surgery was performed to improve the patient’s symptoms. In addition to maximal tumor resection, pulmonary artery valve replacement, artificial vessel replacement, and right total pneumonectomy were performed. Based on the assessment performed using the specimen collected perioperatively, a pathological diagnosis of angiosarcoma of the right pulmonary artery was made. The patient was discharged on postoperative day 17 with a good postoperative course. However, because of dyspnea, she was readmitted to the hospital on day 85. Tumor recurrence was noted, and chemotherapy was initiated. The patient developed cardiac failure and died on postoperative day 119. A pathological postmortem examination was performed. Metastatic lesions were found in the pericardial sac, left lung, right chest wall and pleura, and mediastinum.

**Conclusions:**

In the present case, postoperative recurrence was observed despite maximal resection of the surrounding tissues with tumor invasion and simultaneous reconstruction. Chemotherapy was initiated but was ineffective. Gene panel testing can help identify novel treatment options for patients with neoplastic diseases without standardized treatment. In addition, preparations should be made before surgery.

## Background

Primary pulmonary artery sarcoma progresses extremely rapidly and has a poor prognosis. This condition is rare, with an incidence rate of 0.001%–0.03%. Furthermore, it has no specific symptoms. By the time symptoms such as dyspnea, hemoptysis, and weight loss are observed, the disease is usually in the advanced stage [1–3].

The median survival durations were 1.5 months in patients who did not receive treatment and 10 months in those who underwent surgical resection. However, complete resection is challenging in most cases [4]. Early and accurate diagnosis and complete tumor resection can only slightly increase the survival rate. Moreover, multimodal treatment combining surgery, chemotherapy, and radiation therapy is significantly more effective in prolonging survival than monotherapy [5]. We report the case of a patient with primary pulmonary artery sarcoma who was admitted to the emergency department because of dyspnea.

## Case presentation

A 54-year-old woman was brought to the emergency department because of dyspnea. She had been experiencing chest pain and dyspnea since the previous month and was undergoing follow-up for suspected pericarditis at a local clinic. On the day of hospitalization, the patient was transported via ambulance because of exacerbation of dyspnea and a decline in oxygenation. Orthopnea was also noted.

**Fig. 1:**
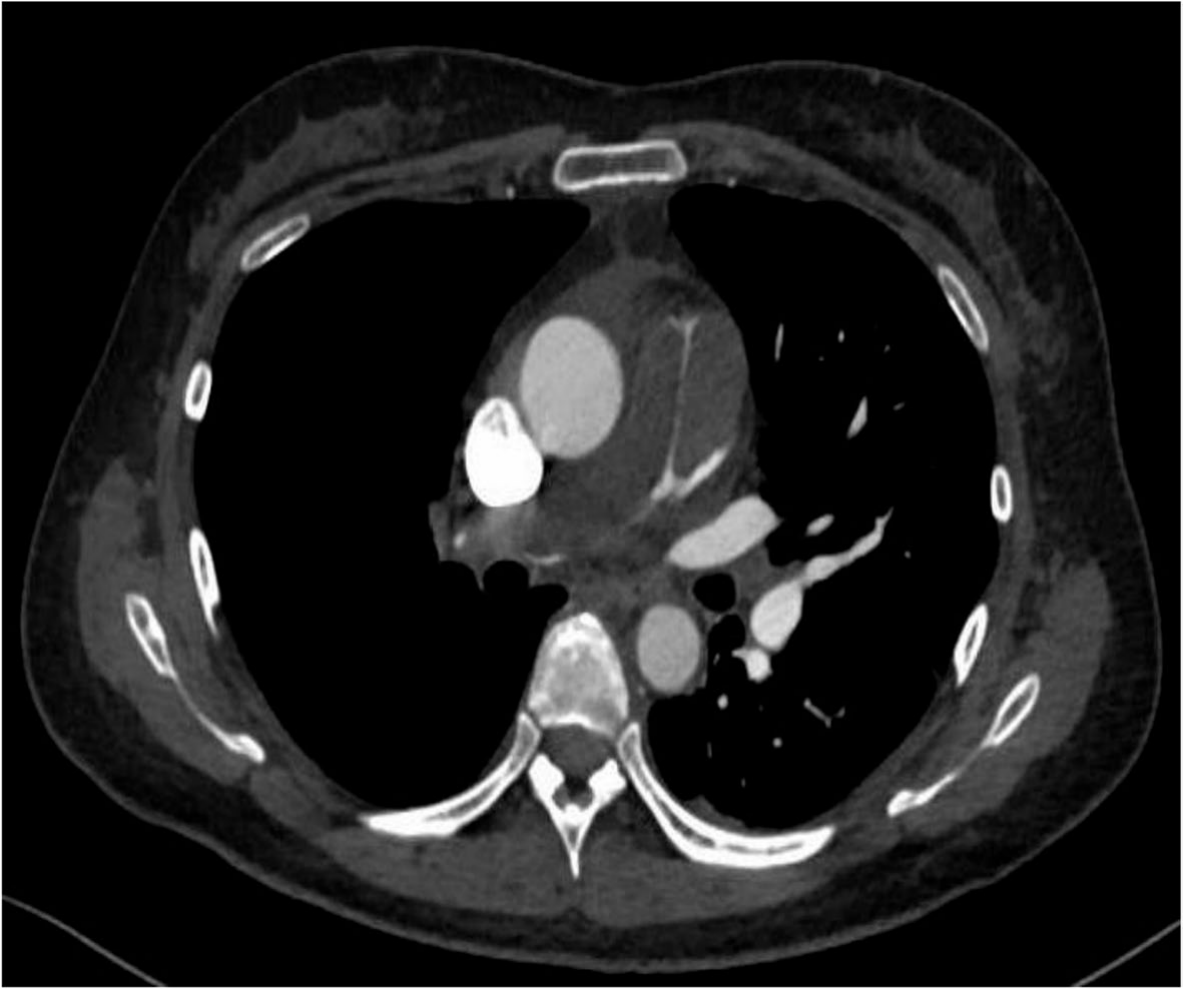
The tumor occluded the primary and right pulmonary arteries

**Fig. 2:**
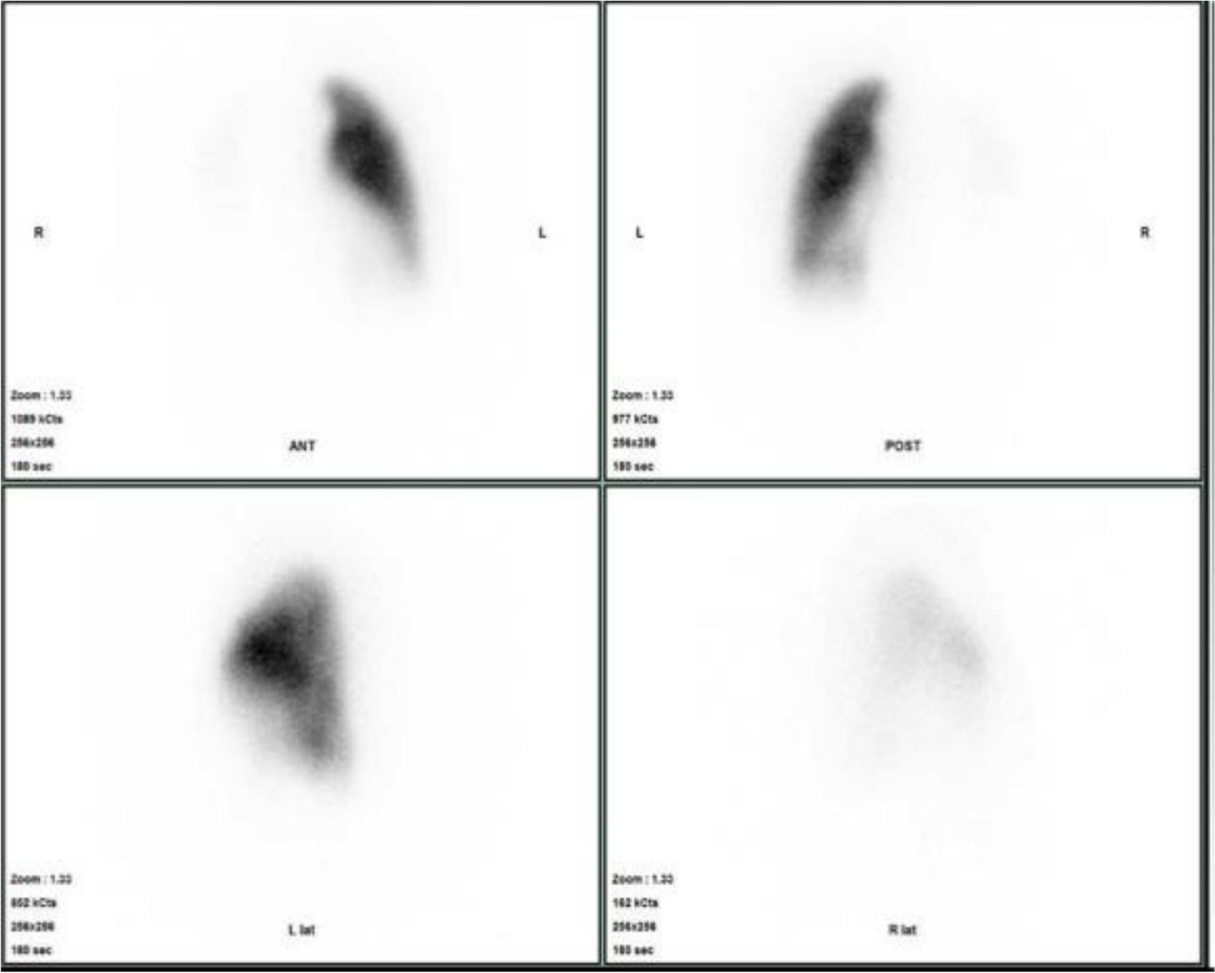
The right lung was barely visible

**Fig. 3:**
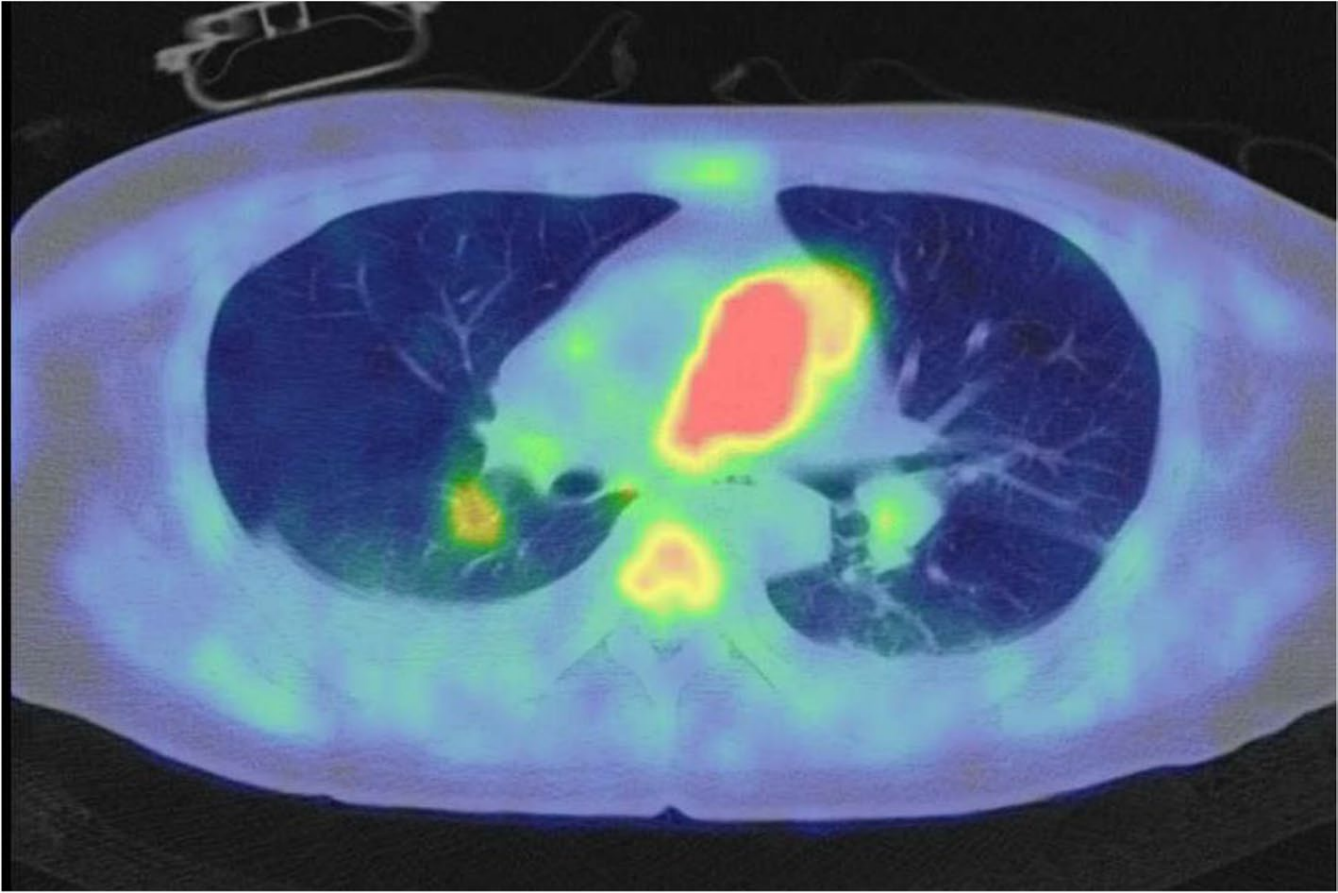
Accumulation from the primary pulmonary artery to the right pulmonary artery (SUVmax = 10.65)

At presentation, the patient’s vital signs were as follows: heart rate, 96 beats/min; blood pressure, 142/112 mmHg; respiratory rate, 24 cycles/min; and oxygen saturation, 98% (O_2_ supplementation: 2 L/min). Heart murmur was not observed. The patient had significant leg edema. Her carcinoembryonic antigen-125 and neuron-specific enolase levels were 61.6 and 32.2, respectively. Furthermore, her tumor marker levels were elevated. The patient’s heart rate was 92 beats/min with sinus rhythm. Chest radiography revealed right secondary arch enlargement, and the cardiothoracic ratio was 62%. Echocardiography revealed severe tricuspid regurgitation, with an elevated tricuspid regurgitation pressure gradient of 96 mmHg, indicating right heart overload. The left ventricle was displaced by the highly enlarged right ventricle. Contrast-enhanced computed tomography scan showed severe stenosis from the pulmonary artery trunk to the right pulmonary artery due to an embolic material. There was no evident thrombus in the deep veins (Fig. 1). On pulmonary scintigraphy, the right lung could be hardly identified (Fig. 2). Positron emission tomography-computed tomography scan revealed accumulation, with an SUVmax of 10.65, in the soft tissue shadow between the primary pulmonary artery trunk and the right pulmonary artery. Other abnormal accumulations indicative of neoplastic lesions or findings suggestive of metastases were not observed (Fig. 3).

Based on the aforementioned data, the most likely cause was pulmonary artery obstruction caused by the tumor. Emergency surgery was performed to relieve dyspnea.

**Fig. 4:**
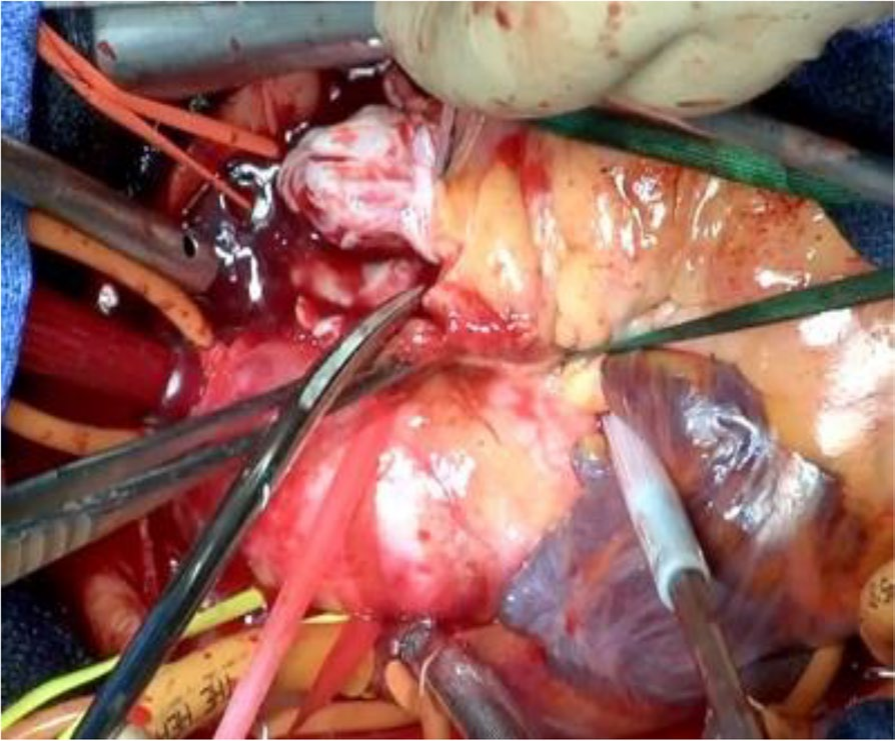
Tumor-filled primary pulmonary artery

**Fig. 5:**
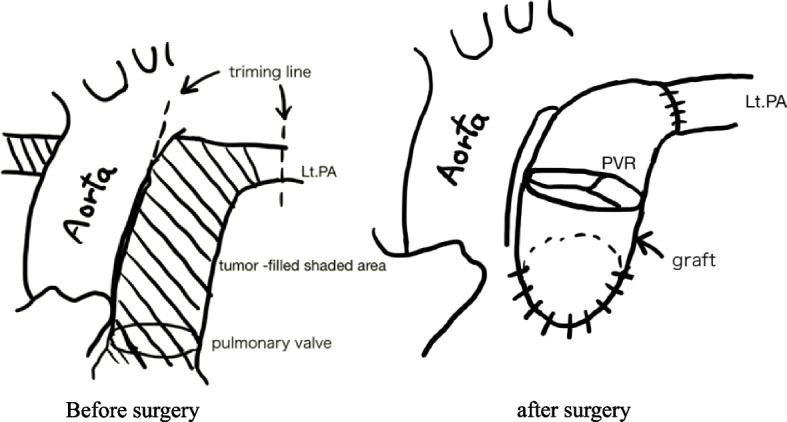
Resection site and vascular graft anastomosis. The shaded area was filled with the tumor. Abbreviations: PA, Pulmonary artery; PVR, Pulmonary Valve Replacement

**Fig. 6:**
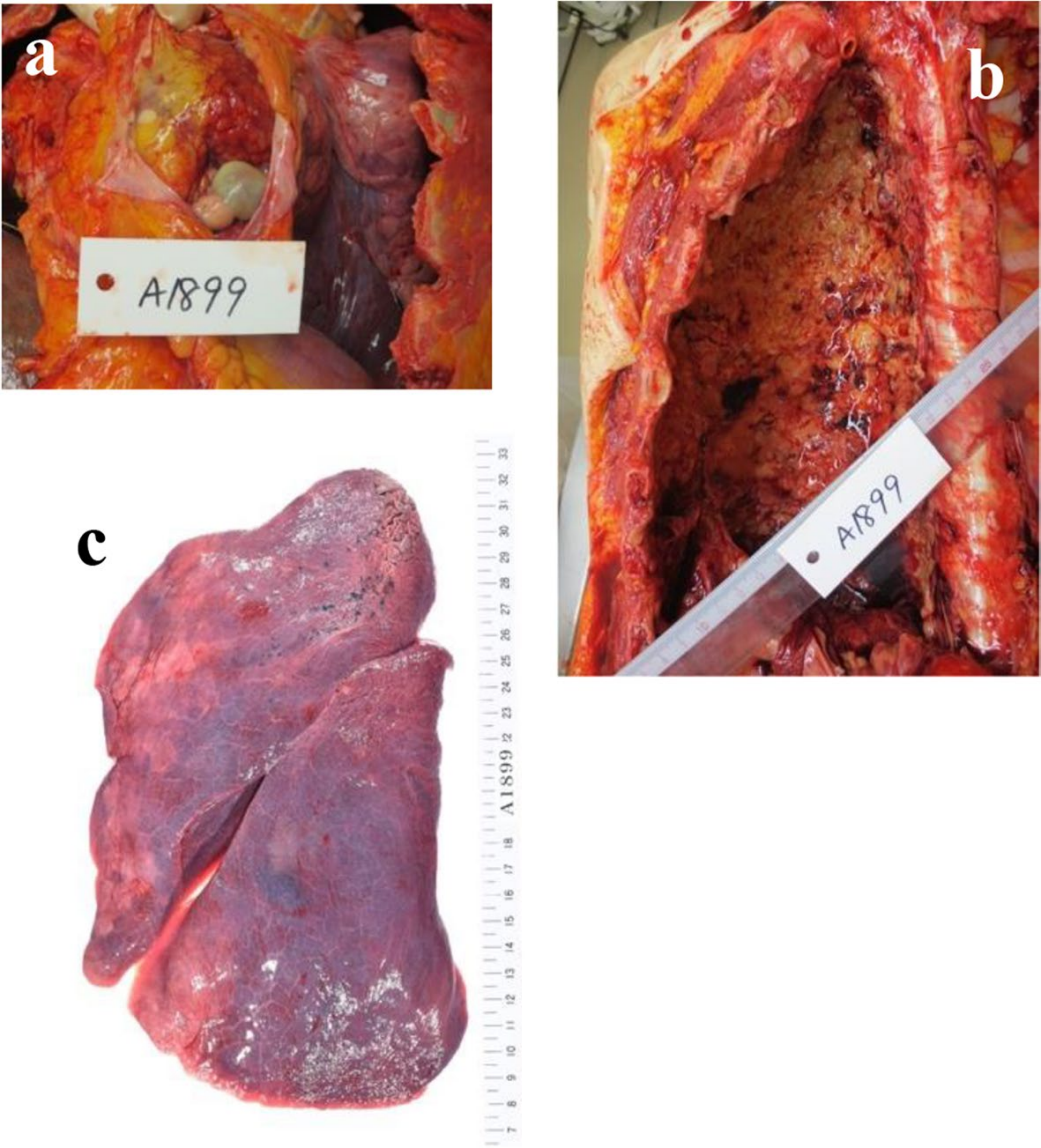
Yellowish-white nodules were scattered within the pericardium. Diffuse tumor growth was observed (**a**). Diffuse tumor growth was noted in the entire right chest wall (**b**). Several small white nodules were found in the left lung (**c**)

Median sternotomy was performed. Subsequently, cardiopulmonary bypass was established by pumping blood into the ascending aorta and removing blood from the superior and inferior vena cava. A vent cannula was inserted from the right superior pulmonary vein into the left ventricle. The main pulmonary artery was incised while the heart was beating. The lumen was filled with the tumor, and the right pulmonary artery was occluded (Fig. 4). The right pulmonary artery was dissected from the bifurcation. Subsequently, the left pulmonary artery was dissected at the normal site where the tumor was not macroscopically visible. On the central side, the tumor extended to the pulmonary artery valve and was resected up to the right ventricular outflow tract where the tumor was not macroscopically visible. There was no evident extravascular invasion observed macroscopically. The pulmonary artery and valve were reconstructed by creating a valved graft by sewing a 19-mm CEPMagnaEASE graft to a 22-mm J-graft (Fig. 5). The central portion of the graft was trimmed in a large tongue-like shape and sutured to prevent stenosis. The patient was weaned from the cardiopulmonary bypass, and percutaneous cardiopulmonary support was resumed. Total right pneumonectomy was performed under the direction of a thoracic surgeon. The entire right pulmonary artery was removed from the transected margins. The patient was easily weaned from the percutaneous cardiopulmonary support device intraoperatively. The surgical duration was 7 h and 37 min, and the cardiopulmonary bypass lasted for 2 h. Postoperatively, the catecholamine dose was reduced relatively easily, and the patient was weaned from the ventilator on the same day. The patient did not exhibit recurrence of heart failure, and her dyspnea improved. She was discharged on postoperative day 17. The pathological diagnosis was angiosarcoma of the right pulmonary artery. The left pulmonary artery and central margin was negative for tumor cells. Complete resection of the tumor was achieved, and no residual tumor was detected on postoperative CT imaging. Consequently, neither adjuvant chemotherapy nor radiation therapy was deemed necessary. On postoperative day 85, the patient was brought to the emergency department because of dyspnea and presented with tumor recurrence. Treatment with paclitaxel was initiated. However, her heart failure progressed, and she died on postoperative day 119. A pathological postmortem examination was performed. Results showed the presence of diffuse yellowish-white tumor nodules and disseminated lesions in the heart. In addition, diffuse tumor growth was observed in the left lung, right chest wall, and thoracic cavity, and the patient exhibited metastatic lesions (Fig. 6).

## Discussion

Primary pulmonary artery sarcoma was first described by Mandelstamm in 1923. This is a rapidly progressive disease with a poor prognosis. It is a rare condition, with an incidence rate of 0.001%–0.03%. Furthermore, its incidence is 1.3 times higher in women than in men, and primary pulmonary artery lesions are more common in individuals in their late 40 s [1, 2, 6].

Malignant tumors account for 25% of primary cardiac tumors, with sarcomas comprising 75% of these cases. Among sarcomas, intimal sarcoma accounts for 50%, followed by angiosarcoma (25%) and undifferentiated sarcoma (20%), with other types such as synovial sarcoma and leiomyosarcoma making up the remainder. Intimal sarcoma predominantly arises in the pulmonary artery or aorta. The prognosis is reported to be 5–9 months for aortic-origin tumors and 13–18 months for pulmonary artery-origin tumors [7]. Intimal sarcoma tends to arise in the intimal layer of the great vessels, particularly the pulmonary artery and aorta. These sarcomas are characterized by intravascular proliferation, ultimately leading to vascular obstruction and distal embolic dissemination. Intimal sarcoma occurs twice as frequently in the pulmonary artery as in the aorta. The tumor often originates in the pulmonary trunk or the right and left pulmonary arteries and frequently extends peripherally along the bloodstream [8]. Intimal sarcoma can also originate from the right ventricular outflow tract or cardiac valves. In such cases, one report indicated a 100% extension into the main pulmonary artery, involvement of the pulmonary valve in 57% of cases, right ventricle in 25%, right pulmonary artery in 67%, and left pulmonary artery in 60% [9]. Approximately 16–25% of patients develop distant lung metastases as a result of tumor embolization [10, 11].

Tumors often originate from the pulmonary artery trunk or right and left pulmonary arteries and spread peripherally to the bloodstream. Thus, the involvement of the pulmonary valve, as observed in this case, is relatively rare. The median survival duration of patients who do not receive treatment is 1.5 months, and most deaths are attributed to right heart failure caused by an increase in right heart load. If surgical resection is performed, the survival duration is 10 months. However, complete resection is challenging in most cases [4].

The symptoms of primary pulmonary artery sarcoma, which include dyspnea, cough, hemoptysis, chest pain, and weight loss, are not specific. Most patients do not present with symptoms until pulmonary artery obstruction occurs. Therefore, the disease is often already severe by the time symptoms are observed [3].

There are no available diagnostic criteria because the specific clinical features of this disease remain unclear. The clinical findings include systolic murmur, jugular vein distension, pulmonary artery murmur, edema, cyanosis, and hepatomegaly [5].

Imaging tests are useful for diagnosis. However, they are also variable and atypical. In addition to the presence of emboli in the pulmonary arteries, nodules in the lungs, infiltrative shadow, ground glass opacity, and pleural effusion are observed [12, 13]. On contrast-enhanced CT, the tumor appears as a heterogeneously enhanced mass, which is helpful in differentiating it from pulmonary thromboembolism. In cases where CT diagnosis is challenging, PET-CT uptake evaluation can provide additional information, while MRI has shown utility for further differentiation when PET-CT is not definitive [14].

Histopathologically, pulmonary artery angiosarcoma is characterized by the proliferation of irregular vascular structures, endothelial cells within the vascular lumen, increased cytoplasm, nuclear atypia, and abnormal mitotic figures. Immunohistochemically, CD31, CD34, and factor VIII-related antigen are often positive [15].

Surgical resection remains the cornerstone of treatment, with complete resection offering a significant improvement in survival. Due to limited evidence for adjuvant therapies, there is no established standard protocol, and treatment decisions are based on individual cases. Adjuvant therapies may be considered in younger patients or those deemed to have a high risk of recurrence even when complete resection is achieved [16].A case was reported where resection and reconstruction from the right ventricular outflow tract to the pulmonary artery bifurcation, along with chemoradiotherapy for residual tumor, resulted in 56 months of disease-free survival [17].

In this case to achieve maximal tumor resection, the surrounding tissues that were infiltrated by the tumor were resected. The intraoperative gross findings and postoperative pathological examination results were negative for margins, suggesting that the tumor was completely resected; however, the tumor recurred and metastasized to multiple organs. Nevertheless, the patient eventually developed recurrence and multiple organ metastasis. In recent years, with gene panel tests, novel treatments for diseases without established treatment methods, as in this case, have been identified. Furthermore, blood tests can be performed. However, they have a lower accuracy than the assessment of excised specimens. In this study, these tests were not performed because the specimens were not stored. Nonetheless, this should be considered in the future.

We reported a case of primary pulmonary artery sarcoma originating from the pulmonary artery and spreading to the pulmonary valve. The tumor was managed with pulmonary valve replacement, pulmonary angioplasty, and right pneumonectomy. There are few reports on cases in which total resection of one lung and pulmonary artery valve replacement were performed in addition to tumor resection, as in this case. Complete tumor resection and postoperative multimodal treatment can improve prognosis. However, gene panel testing should be performed to expand treatment options.

## Data Availability

Not applicable.
